# Identification and validation of a DNA methylation-driven gene-based prognostic model for clear cell renal cell carcinoma

**DOI:** 10.1186/s12864-023-09416-z

**Published:** 2023-06-07

**Authors:** Qiong Deng, Ye Du, Zhu Wang, Yeda Chen, Jieyan Wang, Hui Liang, Du Zhang

**Affiliations:** 1 Department of Urology, Affiliated Longhua People’s Hospital, Southern Medical University, Shenzhen, 518109 China; 2 College of Basic Medicine, Southern Medical University, Guangzhou, 510515 China; 3 Central Laboratory, Affiliated Longhua People’s Hospital, Southern Medical University, Shenzhen, 518109 China; 4 Genome Analysis Laboratory of the Ministry of Agriculture, Agricultural Genomics Institute at Shenzhen, Chinese Academy of Agricultural Sciences, No 7, Pengfei Road, Dapeng New District, Shenzhen, 518120 China

**Keywords:** Clear cell renal cell carcinoma, DNA methylation, Reduced representation bisulfite sequencing, Prognostic model

## Abstract

**Background:**

Clear cell renal cell carcinoma (ccRCC) is a malignant tumor with heterogeneous morphology and poor prognosis. This study aimed to establish a DNA methylation (DNAm)-driven gene-based prognostic model for ccRCC.

**Methods:**

Reduced representation bisulfite sequencing (RRBS) was performed on the DNA extracts from ccRCC patients. We analyzed the RRBS data from 10 pairs of patient samples to screen the candidate CpG sites, then trained and validated an 18-CpG site model, and integrated the clinical characters to establish a Nomogram model for the prognosis or risk evaluation of ccRCC.

**Results:**

We identified 2261 DMRs in the promoter region. After DMR selection, 578 candidates were screened, and was correspondence with 408 CpG dinucleotides in the 450 K array. We collected the DNAm profiles of 478 ccRCC samples from TCGA dataset. Using the training set with 319 samples, a prognostic panel of 18 CpGs was determined by univariate Cox regression, LASSO regression, and multivariate Cox proportional hazards regression analyses. We constructed a prognostic model by combining the clinical signatures. In the test set (159 samples) and whole set (478 samples), the Kaplan–Meier plot showed significant differences; and the ROC curve and survival analyses showed AUC greater than 0.7. The Nomogram integrated with clinicopathological characters and methylation risk score had better performance, and the decision curve analyses also showed a beneficial effect.

**Conclusions:**

This work provides insight into the role of hypermethylation in ccRCC. The targets identified might serve as biomarkers for early ccRCC diagnosis and prognosis biomarkers for ccRCC. We believe our findings have implications for better risk stratification and personalized management of this disease.

**Supplementary Information:**

The online version contains supplementary material available at 10.1186/s12864-023-09416-z.

## Background

Renal cell carcinoma (RCC) is the most prevalent subtype of renal cancer with more than 400,000 new cases detected every year, and it is the second leading cause of death due to urological malignancy [1, 2]. 70–75% of the RCC is clear cell RCC (ccRCC), which is the primary histologic type of RCC and accounts for most deaths due to renal cancer [3, 4]. Although remarkable progress has been made in ccRCC treatment in recent years, the overall prognosis of ccRCC is poor, particularly for patients with advanced-stage ccRCC [5, 6]. The clinical course of patients with ccRCC is heterogeneous; some live for decades without requiring any treatment while other patients experience rapid disease progression. The clinicopathological risk factors and their integrated systems, such as the Mayo Clinic stage, size, grade, and necrosis (SSIGN) score, have greatly improved the prognostic accuracy [7, 8]. However, due to genetic heterogeneity, clinical parameters based on morphology and immunohistochemistry are inadequate to predict the prognosis of ccRCC patients [9, 10]. With growing insights into the molecular biological mechanism of ccRCC, molecular biomarkers which could be able to reflect the biological behavior of ccRCC are believed to add prognostic value to traditional clinical characteristics [11, 12]. Therefore, there is an urgent need to develop reliable genetic prognostic models.

Epigenetic modifications do not change the DNA sequence and could be attributed to heritable alterations [13, 14]. Epigenetic modifications directly impact the function of the human genome by controlling DNA packaging. DNA methylation (DNAm) is one of the major epigenetic modifications, has a crucial role in maintaining gene transcription and genome stability. DNA methylation is reported in numerous studies that has an important effect on carcinogenesis, mainly occurs at the cytosine-phosphate-guanine (CpG) dinucleotide [15]. Regarding the methylation levels, CpG islands are generally hypomethylated, except that a small number randomly distributed [15]. Aberrant DNAm status, including hypomethylation of oncogenes and hypermethylation of tumor suppressor genes, is an important carcinogenic event [16, 17]. Aberrant promoter methylation may lead to lower gene expression or complete silencing of tumor suppressor and caretaker genes [18]. It was shown that DNAm could be used for developing diagnostic and prognostic biomarkers and targeted therapies in The Cancer Genome Atlas (TCGA) project and other studies [19]. Hence, studies were foucus on identifying DNAm-driven genes and investigating their molecular mechanisms, which might be of greatly help in understanding the biological characteristics of ccRCC. Moreover, due to the relatively stable and potentially reversible therapeutic attributes of DNAm in multiple types of cancers, aberrant DNAm have a promising prospective utility as targets for developing robust biomarkers for clinical decision-making [20–22].

Genetically, DNAm is reported to play a significant role during the pathogenesis of ccRCC, which involves both epigenetic and genetic alterations and is characterized by a complex biological disorder [12, 23, 24]. Intriguingly, high stage and grade of ccRCC cases were correlated with increased promoter hypermethylation frequency, and due to hypermethylation of enhancers, the expression of numerous tumor suppressor genes were inihibted, in turn to lead a growth biological activity of progressive tumor cell with respect to ccRCC development [25].

Although numerous studies have focused on the relationship between aberrant DNAm status and ccRCC outcomes, prognostic models based on DNAm-driven genes have rarely been reported with respect to ccRCC. In this study, we profiled the methylome of ccRCC and adjacent tissues from 10 patients using reduced representation bisulfite sequencing (RRBS). We analyzed the global and local methylation divergence and its functional relevance in tumorigenesis. By integrating the DNAm data of 478 patients from the TCGA database, we developed and validated a practical and reliable prognostic model for ccRCC. Our findings will further improve prognosis prediction and individualized treatment for patients with ccRCC.

## Methods

### Patients and study design

As shown in Supplementary Fig. 1, the study procedure included the discovery, training, and validation stages. Briefly, we analyzed the RRBS data from 10 pairs of patient samples (normal vs. tumor) to screen the original candidate CpG sites, then trained and validated an 18-CpG site model, and integrated the clinical characters to establish a Nomogram model for the prognosis or risk evaluation of ccRCC.

As mentioned above, ten patients with ccRCC were recruited for the study. Sample 2 was excluded because of its low correlation with the other samples (Table 1). The diagnosis of ccRCC was based on pathological findings (Supplementary Table 1). The samples were obtained from ccRCC patients who underwent partial nephrectomy to remove cancerous tissue at The People’s Hospital of Longhua. All samples were analyzed by an expert pathologist from the Department of Pathology and kept frozen until used for DNA extraction. Written informed consent was obtained from all participants, and the clinical protocol was reviewed and approved by the Ethics Committee of The People’s Hospital of Longhua, Shenzhen, China. Patients with a follow-up time of less than 30 days were excluded from the survival analysis.

**Table 1:** Detailed information of the recruited ccRCC patients

No	Gender	Age	Location	Size
S1	Male	39	Left	2.0*2.0 cm
S2	Male	45	Right	1.5*2.0 cm
S3	Male	36	Left	1.2*1.5 cm
S4	Male	30	Left	1.5*2.0 cm
S5	Male	33	Right	5.0*6.0 cm
S6	Male	57	Right	3.0*3.0 cm
S7	Male	48	Right	2.0*3.0 cm
S8	Male	53	Right	2.0*3.0 cm
S9	Male	25	Left	2.0*2.0 cm
S10	Male	45	Left	5.0*6.0 cm

A total of 478 patients with ccRCC were obtained through the TCGA project (Table 2). Since it is still disputed whether hypertension, smoking, and obesity are independent risk factors for renal cancer [26, 27], these clinical signatures were not included in this study.

**Table 2:** Clinical and pathological features of the 478 patients from TCGA-KIRC

Features	TCGA-KIRC dataset (*N* = 478)
**Gender**	
Female	164 (34.3%)
Male	314 (65.7%)
**Age**	
Mean (SD)	62.2 (11.6)
Median [Min, Max]	61.9 [26.6, 88.8]
**Neoplasm histologic grade**	
G1	8 (1.7%)
G2	193 (40.4%)
G3	189 (39.5%)
G4	83 (17.4%)
GX	5 (1.0%)
**TNM stage**	
Not reported	2 (0.4%)
Stage I	209 (43.7%)
Stage II	47 (9.8%)
Stage III	124 (25.9%)
Stage IV	96 (20.1%)
**pT stage**	
T1	216 (45.2%)
T2	65 (13.6%)
T3	182 (38.1%)
T4	15 (3.1%)
**Overall Survival time (days)**	
Mean (SD)	1261 (996)
Median [Min, Max]	1091.5 [3, 4537]

### RRBS library construction and sequencing

The renal tissue methylation profiles were studied using the RRBS method by Shenzhen E-Gene Biotechnology Co. Ltd. (Shenzhen, China) with modifications according the lab situation [28, 29]. Genomic DNA was extracted from fresh frozen tissue. The samples were homogenized in lysis buffer consisting of 100 mM Tris–HCl (pH 8.5), five mM EDTA, 0.2% SDS, and 200 mM NaCl. Proteinase K was added at a final concentration of 300 μg/ml. The samples were incubated overnight at 55 °C to ensure that the genomic DNA was dissociated entirely from any DNA-binding proteins. After digestion, the genomic DNA was extracted using a genomic DNA extraction kit, according to the manufacturer’s instructions (AllPrep DNA/RNA Mini Kit, Qiagen, USA). DNA quality and quantity were assessed using a NanoDrop spectrophotometer and 0.8% agarose gel electrophoresis.

For each sample [30], 1 μg of genomic DNA was digested overnight using 40 units of MspI (New England Biolabs). The digested DNA was end-repaired and adenylated in a 50 μl reaction consisting of 10 U of exo-Klenow fragments (Enzymatics) and 2 μl each of dGTP (1 mM), dATP (10 mM), and methylated dCTP (1 mM). The reaction was incubated for 30 min at 30 °C and then for another 30 min at 37 °C. The methylated Illumina adapters were ligated to the adenylated DNA fragments in a 20 μl reaction containing 2 μl of concentrated T4 ligase (Enzymatics) at room temperature for 15 min. The ligation products were gel-selected for fragments with insertion sizes of 40–120 bp and 120–220 bp. Bisulfite treatment was conducted using the EZ DNA Methylation Kit (Zymo Research) according to the manufacturer’s protocol. The final libraries were generated using 5 μl of bisulfite converted template in a 14-cycle PCR amplification system using PfuTurbo Cx Hotstart DNA Polymerase (Agilent Technologies) and sequenced using an Illumina Xten with a paired-end 150 bp strategy.

### RRBS data analysis

All the computational R scripts used for data processing and analysis available as Supplementary file 3. Briefly, we removed low-quality reads by TrimGalore. Adapter contamination was removed by Cutadapt (version 1.9) [31]. The reference genome (hg38) and the corresponding annotation files were obtained from the University of California Santa Cruz (UCSC) database. Clean reads were aligned to the reference genome and called the single base resolution methylation level using BSMAP (version 2.73) [32]. The commonly covered CpG sites with sequencing depths ≥ 5 × in all the nine samples were screened for global correlation and cluster analysis among the samples. To identify differentially methylated regions (DMRs) between the two groups, we used metilene (version 0.2–6) [33] with the following criteria: distance between two neighboring candidate CpG sites ≤ 300 bp, CpG sites ≥ 5, methylation level difference > 0.1, and q-value < 0.05 using the Benjamini Hochberg method [34]. For DMRs annotation, the promoter region was defined as the 2-kb upstream sequence and the 0.5-kb downstream sequence of the transcription start site. The gene body region was defined as the 0.5-kb downstream sequence from the transcription start site to the transcriptional termination site. If the gene harbored one or more DMR, of which > 50% bases overlapped with the gene’s promoter or gene body, it was identified as a differentially methylated gene (DMG) [35].

### Construction and validation of the DMR-based prognostic model

#### Stepwise screening of CpG sites

DMRs with a false discovery rate (FDR) q-value < 0.01 and a methylation difference > 0.25 located in the promoter region were selected for further integrative analysis with the TCGA data. The CpG sites in these DMRs from the 450 k microarray were considered candidate CpGs for constructing the prognostic model.

#### Prognostic model

A total of 478 clinical samples and 450 K microarray data of the The Cancer Genome Atlas Kidney Renal Clear Cell Carcinoma (TCGA-KIRC) cohort were downloaded from UCSC Xena. To construct a methylation-based risk prognostic model, we randomly divided these 478 samples into the training set (70%, 319 samples) and test set (30%, 159 samples). To train the model, candidate CpGs significantly associated with prognosis were identified using univariate Cox regression, least absolute shrinkage and selection operator (LASSO) regression (glmnet R package), and a stepwise multivariate Cox regression analyses in the TCGA training set. The linear combination of the regression coefficient derived from the multivariate Cox regression model with a tenfold cross validation process for 5 times was used to generate the prognostic risk score. Based on the risk score, ccRCC patients were divided into the high-risk and low-risk groups through an appropriate cutoff point determined by the survival R package. The log-rank test and Kaplan–Meier (KM) survival curves were used to evaluate the survival differences between the high-risk and low-risk patients. Time-dependent receiver operating characteristic (ROC) curves were employed to measure the predictive performance using the surviva1 ROC R package [36]. The risk score of all validation cohorts was calculated using the same formula in the TCGA training cohort. The cutoff values of the TCGA validation cohort and the whole TCGA cohort were the same as those of the TCGA training cohort. Univariate and multivariate Cox regression analyses were performed to determine whether the prognostic model was independent of traditional clinical features of ccRCC (including age, gender, histologic grade, and pathologic stage). The statistical significance level was set at 0.05. Hazard ratios (HRs) with confidence intervals (95% CIs) were also calculated.

### Construction and evaluation of the nomogram model

The independent prognostic clinicopathological factors selected by the univariate and multivariate Cox regression analyses and the 18-CpG panel-based risk score were integrated to construct a nomogram through the RMS R package [37]. KM survival analysis and time-dependent ROC curves were used to measure the predictive performance of the Nomogram. In addition, the calibration of overall survival (OS) probability at different time points (1, 3, 5, and 10 years) was assessed using the Hosmer–Lemeshow test.

### Protein–protein interaction network analysis

The 18 dmCpG sites were annotated to functional genes (DMGs) using the corresponding 450 K annotation file downloaded from Illumina official website (https://webdata.illumina.com/downloads/productfiles/methylationEPIC/infinium-methylationepic-v-1-0-b4-manifest-file-csv.zip). Protein–protein interaction analysis among these genes was conducted by GeneMANIA (http://genemania.org/).

### Gene set function analysis

Gene Ontology (GO) and Kyoto Encyclopedia of Genes and Genomes (KEGG) functional enrichment analyses of the DMGs were performed using the AllEnricher software with default parameters [38]. GO terms or KEGG pathways with a *P*-value < 0.05 were considered significantly enriched functions.

## Results

### RRBS sequencing quality control

**Fig. 1:**
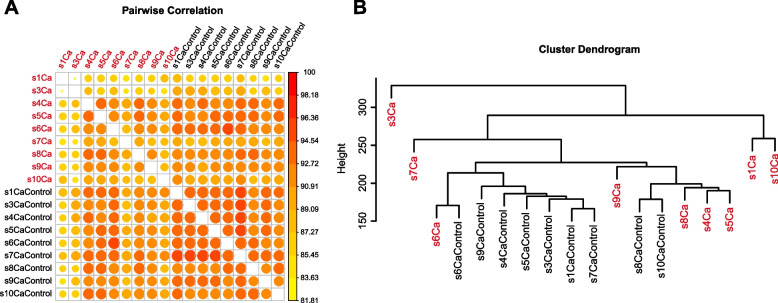
Genome-wide methylation differences among samples. **A** Correlation of CpG methylation rates among samples. A larger circle area and darker color indicate a higher correlation. **B** Cluster dendrogram. According to Euclidean distance, samples were clustered using the hclust function of R. Ward.d2, the minimum variance method, was selected as the clustering method

The flowchart of this study is shown in Supplementary Fig. 1. We analyzed the RRBS data from 10 pairs of patient samples (normal vs. tumor) to screen the original candidate CpG sites, then trained and validated an 18-CpG site model, and integrated the clinical characters to establish a Nomogram model for the prognosis or risk evaluation of ccRCC. The summary of the RRBS sequencing is shown in Table 3. The mapping rate represents the proportion of mapped reads in clean reads. The RRBS sequencing data were analyzed pairwise to determine differentially hypermethylated and hypomethylated CpG sites. The average genome-wide methylation levels of total C, CG, CHG, and CHH are shown in Table 4. CG methylation was the dominant form of methylation among the samples. The correlation analysis of CpG methylation (Fig. 1A) revealed a good correlation of global CpG methylation among the samples. The cluster dendrogram revealed some expected heterogeneity among the tumor samples (Fig. 1B).

**Table 3:** Mapping summary and methylation on C and CpG sites

ID	Raw reads num	Clean reads num	Mapping rate (%)	C ≥ 5 × depth	C ≥ 5 × coverage%	CG ≥ 5 × depth	CG ≥ 5 × coverage%
S1 Ca	70610280	69541152	90.53%	18.4549	4.27607	20.4197	12.3206
S1 Control	76774544	75846068	89.03%	19.5327	3.98545	21.4912	11.708
S2 Ca	76406862	75205782	91.56%	19.0952	4.40915	20.8279	12.7017
S2 Control	67661438	66824480	89.38%	17.7763	4.07378	18.9796	11.6961
S3 Ca	82224792	81161958	88.79%	22.1614	4.01877	24.2684	11.6027
S3 Control	55061390	54403478	90.96%	15.5052	3.73298	17.209	10.9062
S4Ca	67110006	66372658	89.84%	18.1324	4.13492	19.5161	11.8676
S4 Control	65713336	65097216	88.51%	18.0754	3.89858	19.6942	11.1201
S5 Ca	67174320	66413802	88.91%	17.8816	3.99566	19.0881	11.5049
S5 Control	59854658	59189972	91.14%	15.9668	3.60568	17.5145	10.6602
S6 Ca	68838368	68250584	87.99%	16.1532	3.91016	17.1773	11.034
S6 Control	67896208	67240612	90.41%	16.5562	4.04057	17.7704	11.6787
S7 Ca	60636066	60021972	88.80%	12.2743	2.70006	14.0251	7.91118
S7 Control	72072566	71210874	92.96%	18.4785	4.55952	20.6071	13.0421
S8 Ca	72406728	71885174	87.35%	20.4828	3.90657	21.2554	11.0308
S8 Control	71739188	71032094	88.84%	17.2208	4.07385	18.8854	11.8001
S9 Ca	73334944	72588248	88.39%	19.6719	4.29837	21.1938	12.1539
S9 Control	65025156	64526486	90.12%	14.7728	3.00057	16.7971	8.49893
S10 Ca	64097306	63440050	88.85%	16.7965	3.60493	18.5089	10.0266
S10 Control	89006670	87925188	91.26%	20.0768	4.50314	23.8406	12.9384

**Table 4:** CG, CHG, CHH average methylation level (> 1X depth)

ID	C	CG	CHG	CHH
S1Ca	0.085313	0.679945	0.01098	0.008996
S1 Control	0.076032	0.679417	0.014491	0.012859
S2 Ca	0.063247	0.564915	0.009541	0.007948
S2 Control	0.073132	0.668604	0.009896	0.008796
S3 Ca	0.063247	0.564915	0.009541	0.007948
S3 Control	0.073132	0.668604	0.009896	0.008796
S4Ca	0.075968	0.645839	0.010519	0.008557
S4 Control	0.081017	0.659898	0.013712	0.012085
S5 Ca	0.074902	0.648588	0.009792	0.007965
S5 Control	0.081529	0.661092	0.014666	0.013155
S6 Ca	0.073316	0.660722	0.010186	0.008449
S6 Control	0.072124	0.675659	0.013316	0.012018
S7 Ca	0.066089	0.689941	0.010362	0.009398
S7 Control	0.068595	0.682751	0.012587	0.011059
S8 Ca	0.058166	0.688835	0.007909	0.007115
S8 Control	0.084331	0.65613	0.01549	0.014059
S9 Ca	0.072952	0.655497	0.010275	0.008498
S9 Control	0.07211	0.673234	0.01269	0.01129
S10Ca	0.07978	0.667015	0.011118	0.009387
S10 Control	0.058454	0.700477	0.010731	0.009895

### Identification of DMRs

**Fig. 2:**
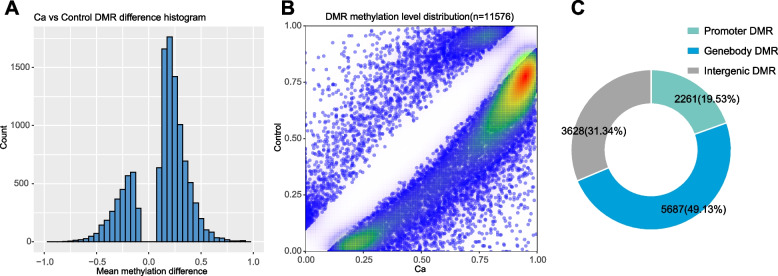
DMR analysis. **A** Mean methylation difference. The X-axis represents the methylation difference. Negative values indicate low methylation levels. Positive values indicate increased methylation levels. The Y-axis represents the number of DMRs within the corresponding abscissa range. **B** DMR methylation distribution density. The X-axis represents the DMR methylation level in the Ca group; the Y-axis represents the DMR methylation level in the control group. The DMR density varies from low to high (white to red). **C** The distribution of DMR on gene elements

To identify the local methylation alterations between the ccRCC and adjacent normal tissues, we performed genome-wide DMR detection. We finally identified 11,576 DMRs (q-value < 0.05) associated with 7948 annotated genes, among which an equivalent proportion of hypermethylated (8528) and hypomethylated (3048) regions was uncovered (Fig. 2A & B).

Promoter DNAm regulates gene expression by binding with transcription factors, particularly those possessing CpG-rich response elements [39]. It was reported that methylation in the promoter region can inhibit gene transcription, while methylation in gene body increases target gene expression. Considering that the transcriptional effects of DNAm are highly dependent on the position of DMRs, and the methylation status of the promoter is always classically negatively associated with mRNA transcription [40, 41], the distribution of DMRs in the genomic locations was investigated. Annotated DMRs were mapped onto the CpG island-related regions and the genic location (promoter, 5’UTR, gene body, and 3’UTR) (Fig. 2C). We performed GO and KEGG pathways analyses to reveal the biological functions of DMGs. The GO and KEGG pathway analyses of DMGs with DMRs in the promoter and gene body regions are shown in Supplementary Fig. 2.

### Construction of the ccRCC prognostic model

After multiple testing adjustments, 578 out of 2261 DMRs, which were located in the promoter regions (5’-UTR, TSS200, TSS1500, and first exon), survived the stringent statistical test (*P*-value < 0.001, methylation difference > 0.25). These 578 sites corresponded to 408 sites in the 450 K microarray (Infinium HumanMethylation450 Bead Chip).

A total of 478 patients with complete clinical information in the TCGA-KIRC data set were included, with 319 samples (70%) in the training set and 159 samples (30%) in the test set. As illustrated in the Fig. 1, univariate Cox regression analysis was conducted to investigate the prognostic value of the methylation levels of 408 differentially methylated CpG (dmCpG) sites using the TCGA training cohort. Seventy four survival-associated dmCpG sites were obtained with the threshold of *P* < 0.001 (Supplementary Table 2).

**Fig. 3:**
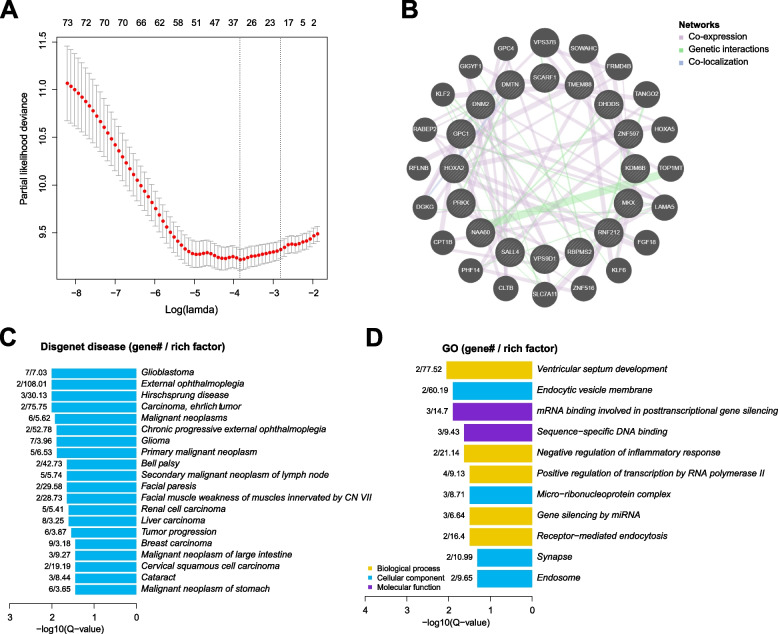
The dmCpG site identification. **A** LASSO regression analysis of CpG sites with tuning parameter selection (lambda). **B** Protein–protein interactions. **C** DisGeNet disease enrichment analysis of 18 dmCpG sites. **D** GO enrichment analysis of 18 dmCpG sites

Next, thirty key dmCpG sites were identified in the LASSO regression analysis with cross-validation, where the regularization parameter was chosen based on tenfold cross-validation (Fig. 3A). Then eighteen candidate dmCpGs sites were screened by the stepwise Cox regression analysis for the construction of the prognostic signature (Table 5). The expression profile and the DNA methylation profile of promoters of 18-CpG corresponding genes were shown in Supplementary Fig. 3, and Supplementary Fig. 4, respectively. Figure 3B shows the protein–protein interaction networks of the 18 dmCpG sites related genes created using GeneMANIA. DisGeNET disease analysis results showed that most genes were significantly enriched in tumor progression of ccRCC, including malignant neoplasms, primary malignant neoplasms, renal cell carcinoma, tumor progression, as well as other types of cancers, such as liver carcinoma, breast carcinoma, and malignant neoplasms of lagre intestine and stomach (Fig. 3C). GO analysis revealed that these genes were involved in nagtive regulation of inflammatory response, positive regulation of transcription by RNA polymerase II, and receptor-mediated endocytosis, and the data suggested that these genes play an essential role in tumorigenesis of ccRCC (Fig. 3D).

**Table 5:** Annotation of the 18 CpG sites in the prognostic model

No	CpG site	CHR	MAPINFO	Ref gene name	Ref gene group	Relation to CpG Island
1	cg17868751	X	3633155	PRKX	TSS1500	S_Shore
2	cg06577005	1	26758228	DHDDS	TSS1500	N_Shore
3	cg17367832	2	241395288	MIR149;PP14571;GPC1	TSS200;Body	Island
4	cg23462514	4	1107585	RNF212	TSS200	Island
5	cg25598840	7	27142618	HOXA2	TSS1500	N_Shore
6	cg03021802	8	21923841	EPB49 (DMTN)	5'UTR	Island
7	cg14947429	10	28036151	MKX	TSS1500	S_Shore
8	cg23067082	12	7073179	MIR141	TSS200	
9	cg18210365	15	65066710	RBPMS2	Body	N_Shore
10	cg01286935	16	89778247	C16orf7 (VPS9D1)	Body	Island
11	cg03933495	16	3493614	NAT15(NAA60);ZNF597	TSS200	S_Shore
12	cg00869668	17	1549012	SCARF1	5’UTR;1stExon	S_Shore
13	cg06941557	17	7757543	KDM6B;TMEM88	3’UTR;TSS1500	Island
14	cg13965612	19	10928696	DNM2;MIR199A1	Body;TSS1500	**–**
15	cg06303238	20	50418959	SALL4	1stExon;5’UTR	Island
16	cg24332577	20	50419248	SALL4	TSS1500	S_Shore
17	cg26728517	20	39319540	**–**	**–**	Island
18	cg21655830	21	44899410	C21orf84 (LINC00313)	TSS1500	**–**

The prognostic model was established using the regression coefficient from the multivariate Cox proportional hazard analysis. The coefficient of the prognostic model was shown in Supplementary Table 3. The risk score of each sample was calculated as follows:

$$\mathrm{Risk score for patients}= {\sum }_{i=1}^{\mathrm{N}}\left(\mathrm{cofficients of each CpGs }\times\upbeta -\mathrm{values of each CpGs}\right)$$

### Training of the prognostic model

**Fig. 4:**
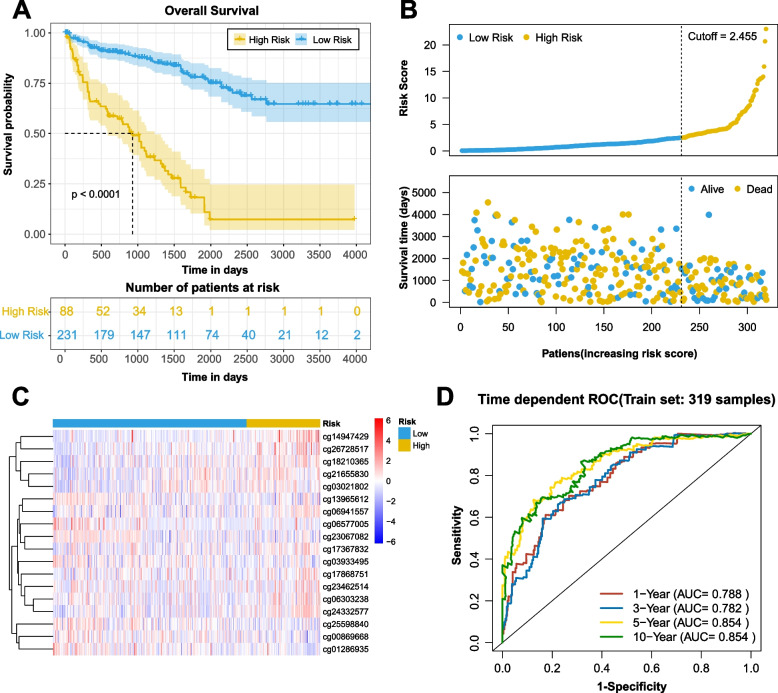
Risk scores in the training set. **A** KM survival curve of patients in the high-risk and low-risk groups. The data are shown as median with the interquartile range. Statistical significance was assessed using Log-rank test. The dotted line shows the statistical significance at 50% survival probability. **B** Rank of calculated risk score and survival status of high-risk and low-risk patients. The dotted line shows the cutoff value to distinguish ccRCC high-risk and low-risk patients. **C** Heat map of methylation levels at 18 CpG sites. **D** The 1-, 3-, 5-, and 10-year ROC curves of risk scores. The sensitivity and specificity of this model were determined by the cutoff value

We examined the distribution of the survival status of all patients. For patients in the TCGA training set, we applied the median risk score (2.455) as the cutoff value, which was computed according to the normalized methylation levels of the 18 CpG sites, to separate the patients into the high-risk and low-risk statuses. The survival analysis of the risk score also revealed the survival probability divergence between the high-risk and low-risk patients (*P* < 0.0001, Fig. 4A and B). The heat map in Fig. 4C shows that the variation direction of the methylation levels of the 18 CpG sites was constant with their coefficients in the prognostic signature. Moreover, 1-, 3-, 5-, and 10-year ROC curves of risk scores were plotted in Fig. 4D, with AUC values of 0.788, 0.782, 0.854, and 0.854, respectively. These results indicated good prognostic prediction efficacy of the 18-CpG site model.

**Fig. 5:**
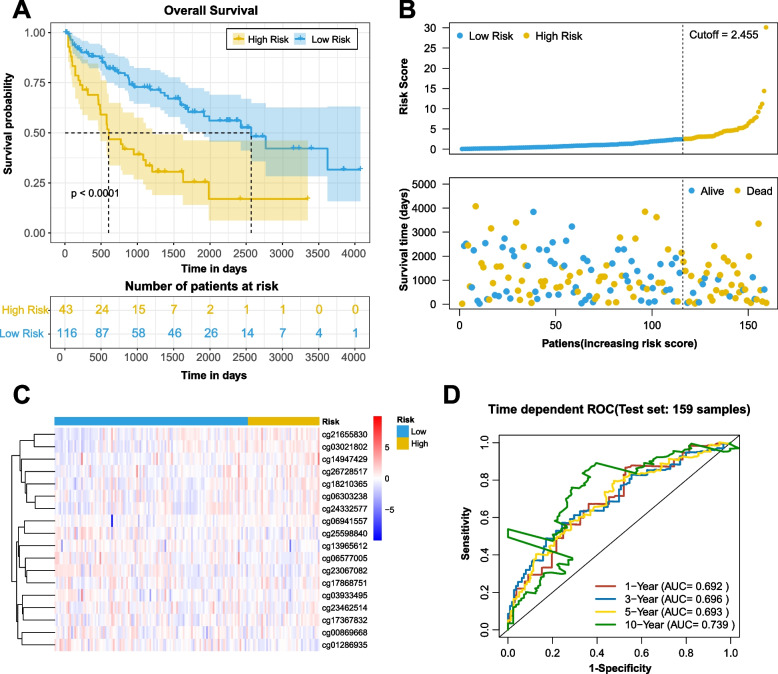
Risk scores in the test set. **A** KM survival curve of patients in the high-risk and low-risk groups. The data are shown as median with the interquartile range. Statistical significance was assessed using Log-rank test. The dotted line shows the statistical significance at 50% survival probability. **B** Rank of calculated risk score and survival status of high-risk and low-risk patients. The dotted line shows the cutoff value to distinguish ccRCC high-risk and low-risk patients. **C** Heat map of methylation levels at 18 CpG sites. **D** The 1-, 3-, 5-, and 10-year ROC curves of risk scores. The sensitivity and specificity of this model were determined by the cutoff value

The prognostic prediction ability of the 18-CpG site model was also validated in patients from the test set, and similar results were obtained (Fig. 5). As shown in Fig. 5A and B, consistent with the above findings, survival analysis on the test cohort showed that the high-risk patients had a particularly unfavorable prognosis than the low-risk patients. Time-dependent ROC curves in Fig. 5D displayed that the 18-site prognostic model had reliable predictive accuracy across the test cohort, with AUC values of 0.692, 0.696, 0.693, and 0.739 revealed by the 1-, 3-, 5-, and 10-year ROC curves, respectively.

### Validation of the prognostic model

Specificity of the model for ccRCC was further tested for ccRCC patients in the TCGA whole cohort. High-risk and low-risk groups was classified based on the same cutoff value (2.455) in the TCGA training cohort. Similar results were shown in supplementary Fig. 5. In supplementary Fig. 5D, the AUC values were 0.753, 0.745, 0.794, and 0.825 for 1-, 3-, 5-, and 10- years, respectively. These results demonstrated that the prognostic model for OS also had a good predictive ability for ccRCC, suggesting that the prognostic model was specifically and strongly correlated with the development and progression of ccRCC.

**Fig. 6:**
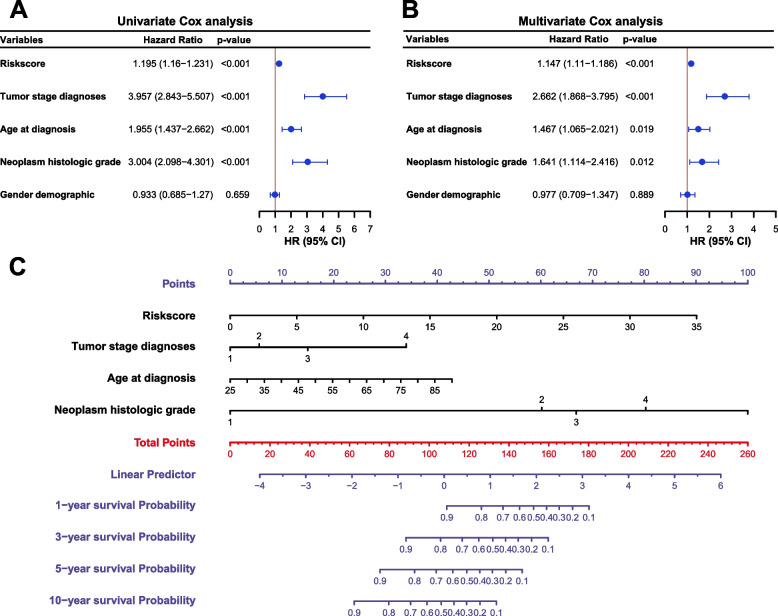
Nomogram based on the prognostic model and clinical characteristics. **A** Univariate and **B** multivariate regression analyses of the prognostic model and clinical characteristics. **C** Nomogram for predicting the probability of 1-, 3-, 5-, and 10-year survival times for ccRCC patients

We performed univariate and multivariate cox analyses to evaluate whether the risk score was an independent prognostic index irrespective of the other clinical features, and the analyses incluled the clinical information of 478 patients in the TCGA data set. As shown in Fig. 6, both of the univariate and multivariate analyses results suggested that tumor, node, metastasis (TNM) stage, age, neoplasm histologic grade, and risk score were independent prognostic indexes (*P* < 0.05), but gender was not an independent prognostic index.

**Fig. 7:**
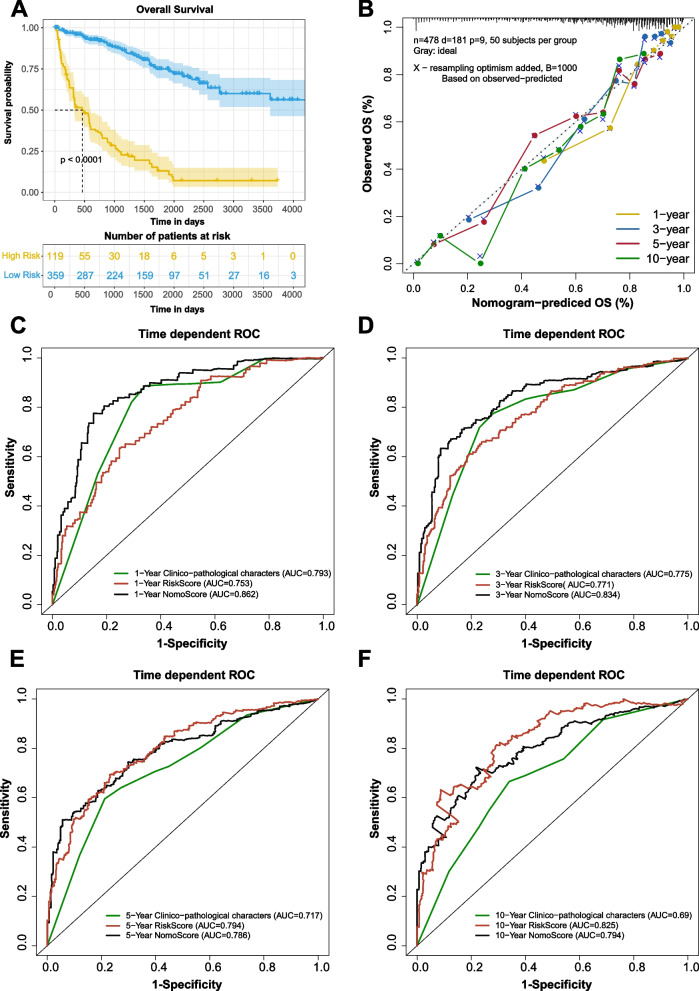
Model calibration and ROC evaluation. **A** KM survival curve of patients in the high-risk and low-risk groups. **B** Nomogram for predicting 1-, 3-, 5-, and 10-year OS for ccRCC patients. ROC curves of clinicopathological characters (green, integrated with tumor stage diagnoses, age and neoplasm histologic grade), RiskScore (red) and NomoScore (black), at 1 (**C**), 3 (**D**), 5 (**E**), and 10 years (**F**)

The OS assay of the prognostic model showed a significant difference in survival probability (*P* < 0.0001, Fig. 7A). The predicted OS performance was good at 1, 3, 5, and 10 years (Fig. 7B). As shown in Fig. 7C, D, at 1, 3 years, the NomoScore explored in our study performed better than the combined clinicopathological characters (tumor stage, histological grade, and age) and the risk score alone. At 5, and 10 years (Fig. 7E, F), the NomoScore and risk score were similar, better and the combined clinicopathological characters. Additionally, the decision curve analyses showed a beneficial effect when integrating the clinicopathological characters with the methylation risk score (Supplementary Fig. 6).

## Discussion

Despite considerable progress has been made in the treatment of ccRCC, ccRCC as one of the most common urological malignancies, still poses a severe public health burden [5, 42]. Similar to the development of other type of cancers, the development of ccRCC is primarily driven by genetic alterations and epigenetic abnormalities [43, 44]. In particular, aberrant methylation is one of the most critical carcinogenic biological processes, especially in ccRCC [23, 45, 46]. The advances in next-generation sequencing technologies and methylation microarray offer emerging opportunities to analyze the genomic profiles and methylome together in an integrated manner [47]. Therefore, it is feasible and promising to identify specific DNAm-driven genes that reflect the biological behavior and predict the prognosis of ccRCC. In this study, we focused on identifying the importance of DNAm-driven genes in ccRCC prognosis. As a result, we constructed and validated an 18 CpG methylation based prognostic model for ccRCC. Furthermore, we established a nomogram by combining the prognostic model with clinical characteristics to help clinicians better manage patients with ccRCC.

Hypomethylation may directly influence karyotypic stability and prompt altered heterochromatic-euchromatic interactions favoring oncogenesis [48]. Moreover, genome-wide DNA hypomethylation is associated with genomic instability, conferring a poor prognosis [49]. RRBS was a widely used cost-efficient method to depict genome-wide DNA methylation alterations in clinical research. Based on the RRBS data, we identified 2261 DMRs in the promoter region. After DMR selection, 578 candidates were screened, and there was correspondence with 408 CpGs in the 450 K array. Using the training set with 319 samples from TCGA, a prognostic panel of 18 CpGs was established. Then univariate Cox regression, LASSO regression, and multivariate Cox proportional hazards regression analyses were performed. By combining the clinical signatures, we aimed to build a DNAm-driven gene-based prognostic model for ccRCC.

Some studies had reported prognostic models for ccRCC. Wang et al. [50] established a three-gene-based prognostic model, and Pan et al. [51] identified a five-gene signature. However, due to lack of internal and external validations in these models, the AUC values of the models are less than 0.7. In our study, an 18 dmCpG-based prognostic model was identified and comprehensively validated. As shown in the results, the KM plot showed significant differences between the high-risk and low-risk patients both in the test set (159 samples) and whole set (478 samples). Besides this, the ROC curve and survival analyses also revealed good performance, with AUC values greater than 0.7. Collectively, all the data suggested a promising model for ccRCC prognosis prediction. This model is an independent and specific indicator of ccRCC prognosis, and it is believed to offer novel prognostic biomarkers and potential treatment targets for ccRCC.

However, this study has several limitations. First, the sequencing was merely carried out in nine male patients. The training and validation of the prognostic model were based only on the in silico and retrospective study of publicly available data. The prediction validation was performed in only one independent cohort. We are planning to perform adequate validation in a larger population-based prospective cohort to strengthen the clinical utility of our findings in the future. Second, the biological functions of 18 CpG site annotated genes should be explored and verified by further experiments, making the methylation-based prognostic model more explainable. With the rapid development of multi-omics technology, we are entering an era of precision medicine. Many biomarkers have been identified based on high throughput sequencing, but very few of them have been identified based on CpG dinucleotide sites. The 18-CpG signature and nomogram explored in our study could guide the clinicians in accurately identifying high-risk ccRCC patients, performing early treatment interventions for ccRCC, and predicting the long-term survival outcomes of ccRCC patients. Nowadays, the detection of CpG sites is more complex and expensive than the detection of gene expression, but hundreds of thousands of CpG sites identified have promising diagnostic and prognostic value, and these CpG sites should be explored further with the development of the detection technology. Moreover, testing of only 18 CpG sites can be a cost-effective routine and may be useful for prognosis prediction in clinical practice. The precise biological mechanisms of ccRCC progression are still unclear, and future functional experiments shoul be emphasized on these mechanisms. In addition, further prospective studies in more medical centers are required to verify the predictive ability and accuracy of this model. Due to the the current model AUC not being high enough, we may try other robust network-based regularization and variable selection for high-dimensional genomic data [52, 53] in future research to improve the robustness and accuracy of the model. In general, despite these shortcomings, we have provided a reliable prognostic model for the clinicians to use while evaluating the individual prognosis of ccRCC patients.

## Conclusions

In this study, We analyzed the RRBS data from patient samples to screen the original candidate CpG sites, then trained and validated an 18-CpG site model using TCGA-KIRC data, and integrated the clinical characters to establish a Nomogram model for the prognosis or risk evaluation of ccRCC.

Based on the result, this novel prognostic model was developed and validated as a practical and reliable predictive tool for patients with ccRCC. In addition, our findings support the notion that aberrant DNAm status is closely associated with oncogenesis and offers potential novel prognostic biomarkers for ccRCC. We believe our findings have implications for better risk stratification and personalized management of this disease.

## Supplementary Information

**Additional file 1:** **Supplementary Figure 1. **Flow diagram of the analysis procedure, including the discovery, training, and validation stages. **Supplementary Figure 2. **DMG function. A, GO and C, KEGG functional enrichment analyses of DMGs in the promoter regions of genes. B, GO and D KEGG functional enrichment analyses of DMGs in the gene body regions. **Supplementary Figure 3. **The expression profile of 18-CpG corresponding genes. **Supplementary Figure 4. **The DNA methylation profile of promoters of 18-CpG corresponding genes. **Supplementary Figure 5. **Risk scores in the whole TCGA cohort. A, KM survival curve of patients in the high-risk and low-risk groups. The data are shown as median with the interquartile range. Statistical significance was assessed using Log-rank test. The dotted line shows the statistical significance at 50% survival probability. B, Rank of calculated risk score and survival status of high-risk and low-risk patients. The dotted line shows the cutoff value to distinguish ccRCC high-risk and low-risk patients. C, Heat map of methylation levels at 18 CpG sites. D, The 1-, 3-, 5-, and 10-year ROC curves of risk scores. The sensitivity and specificity of this model were determined by the cutoff value. **Supplementary Figure 6. **Decision curve analyses for overall survival predictions.The colored lines indicate the net benefit of using the model with the combined clinicopathological characters (red), methylation RiskScore (green) and the NomoScore (black). The assumptions that all patients will be alive and that no patients will be dead are represented by grey and black lines, respectively.

**Additional file 2:** **Supplementary Table 1. **Detailed information of the recruited ccRCCpatients. **Supplementary Table 2. **The univariate Cox regression analysis. **Supplementary Table 3. **The coefficient of the 18 CpG sites. 

**Additional file 3**.

## Data Availability

The datasets generated and analysed during the current study are available in the SRA BioProject database (ID: PRJNA932555, website link: http://www.ncbi.nlm.nih.gov/bioproject/932555).

## Abbreviations

RCC: Renal cell carcinoma
ccRCC: Clear cell renal cell carcinoma
DNAm: DNA methylation
TCGA: The cancer genome atlas
CpG: Cytosine-phosphate-guanine
AUC: Area under the curve
RRBS: Reduced representation bisulfite sequencing
DMRs: Differentially methylated regions
DMGs: Differentially methylated genes
FDR: False discovery rate
LASSO: Least absolute shrinkage and selection operator
KM: Kaplan-Meier
ROC: Receiver operating characteristic
HR: Hazard ratio
OS: Overall survival
GO: Gene ontology
KEGG: Kyoto encyclopedia of genes and genomes
