# Supplementary material for: Identification and validation of a DNA methylation-driven gene-based prognostic model for clear cell renal cell carcinoma
Source: BMC Genomics. 2023 Jun 7;24:307. doi: 10.1186/s12864-023-09416-z (PMC10249168; doi:10.1186/s12864-023-09416-z)
Supplement: Supplementary file 1 — Additional file 1: Supplementary Figure 1. Flow diagram of the analysis procedure, including the discovery, training, and validation stages. Supplementary Figure 2. DMG function. A, GO and C, KEGG functional enrichment analyses of DMGs in the promoter regions of genes. B, GO and D KEGG functional enrichment analyses of DMGs in the gene body regions. Supplementary Figure 3. The expression profile of 18-CpG corresponding genes. Supplementary Figure 4. The DNA methylation profile of promoters of 18-CpG corresponding genes. Supplementary Figure 5. Risk scores in the whole TCGA cohort. A, KM survival curve of patients in the high-risk and low-risk groups. The data are shown as median with the interquartile range. Statistical significance was assessed using Log-rank test. The dotted line shows the statistical significance at 50% survival probability. B, Rank of calculated risk score and survival status of high-risk and low-risk patients. The dotted line shows the cutoff value to distinguish ccRCC high-risk and low-risk patients. C, Heat map of methylation levels at 18 CpG sites. D, The 1-, 3-, 5-, and 10-year ROC curves of risk scores. The sensitivity and specificity of this model were determined by the cutoff value. Supplementary Figure 6. Decision curve analyses for overall survival predictions.The colored lines indicate the net benefit of using the model with the combined clinicopathological characters (red), methylation RiskScore (green) and the NomoScore (black). The assumptions that all patients will be alive and that no patients will be dead are represented by grey and black lines, respectively. [file 12864_2023_9416_MOESM1_ESM.zip › Supplementary Figure2.pdf]

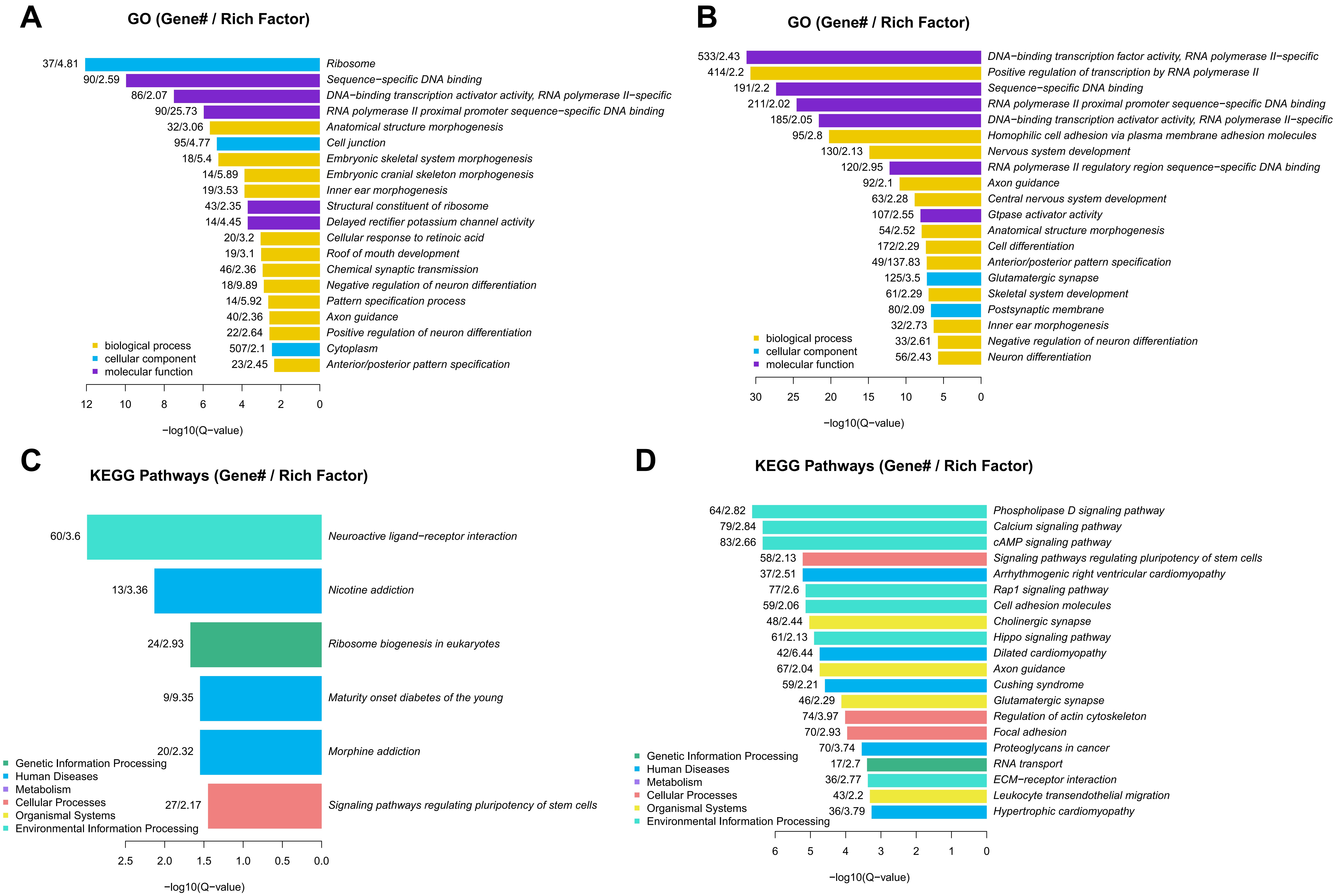
A
GO (Gene# / Rich Factor)
37/4.81 Ribosome
90/2.59 Sequence−specific DNA binding
86/2.07 DNA−binding transcription activator activity, RNA polymerase II−specific
90/25.73 RNA polymerase II proximal promoter sequence−specific DNA binding
32/3.06 Anatomical structure morphogenesis
95/4.77 Cell junction
18/5.4 Embryonic skeletal system morphogenesis
14/5.89 Embryonic cranial skeleton morphogenesis
19/3.53 Inner ear morphogenesis
43/2.35 Structural constituent of ribosome
14/4.45 Delayed rectifier potassium channel activity
20/3.2 Cellular response to retinoic acid
19/3.1 Roof of mouth development
46/2.36 Chemical synaptic transmission
18/9.89 Negative regulation of neuron differentiation
14/5.92 Pattern specification process
40/2.36 Axon guidance
22/2.64 Positive regulation of neuron differentiation
507/2.1 Cytoplasm
23/2.45 Anterior/posterior pattern specification
biological process
cellular component
molecular function
12 10 8 6 4 2 0
−log10(Q−value)
B
GO (Gene# / Rich Factor)
533/2.43 DNA−binding transcription factor activity, RNA polymerase II−specific
414/2.2 Positive regulation of transcription by RNA polymerase II
191/2.2 Sequence−specific DNA binding
211/2.02 RNA polymerase II proximal promoter sequence−specific DNA binding
185/2.05 DNA−binding transcription activator activity, RNA polymerase II−specific
95/2.8 Homophilic cell adhesion via plasma membrane adhesion molecules
130/2.13 Nervous system development
120/2.95 RNA polymerase II regulatory region sequence−specific DNA binding
92/2.1 Axon guidance
63/2.28 Central nervous system development
107/2.55 Gtpase activator activity
54/2.52 Anatomical structure morphogenesis
172/2.29 Cell differentiation
49/137.83 Anterior/posterior pattern specification
125/3.5 Glutamatergic synapse
61/2.29 Skeletal system development
80/2.09 Postsynaptic membrane
32/2.73 Inner ear morphogenesis
33/2.61 Negative regulation of neuron differentiation
56/2.43 Neuron differentiation
biological process
cellular component
molecular function
30 25 20 15 10 5 0
−log10(Q−value)
C
KEGG Pathways (Gene# / Rich Factor)
60/3.6 Neuroactive ligand−receptor interaction
13/3.36 Nicotine addiction
24/2.93 Ribosome biogenesis in eukaryotes
9/9.35 Maturity onset diabetes of the young
20/2.32 Morphine addiction
27/2.17 Signaling pathways regulating pluripotency of stem cells
Genetic Information Processing
Human Diseases
Metabolism
Cellular Processes
Organismal Systems
Environmental Information Processing
2.5 2.0 1.5 1.0 0.5 0.0
−log10(Q−value)
D
KEGG Pathways (Gene# / Rich Factor)
64/2.82 Phospholipase D signaling pathway
79/2.84 Calcium signaling pathway
83/2.66 cAMP signaling pathway
58/2.13 Signaling pathways regulating pluripotency of stem cells
37/2.51 Arrhythmogenic right ventricular cardiomyopathy
77/2.6 Rap1 signaling pathway
59/2.06 Cell adhesion molecules
48/2.44 Cholinergic synapse
61/2.13 Hippo signaling pathway
42/6.44 Dilated cardiomyopathy
67/2.04 Axon guidance
59/2.21 Cushing syndrome
46/2.29 Glutamatergic synapse
74/3.97 Regulation of actin cytoskeleton
70/2.93 Focal adhesion
70/3.74 Proteoglycans in cancer
17/2.7 RNA transport
36/2.77 ECM−receptor interaction
43/2.2 Leukocyte transendothelial migration
36/3.79 Hypertrophic cardiomyopathy
Genetic Information Processing
Human Diseases
Metabolism
Cellular Processes
Organismal Systems
Environmental Information Processing
6 5 4 3 2 1 0
−log10(Q−value)
