# Supplementary material for: Identification and validation of a DNA methylation-driven gene-based prognostic model for clear cell renal cell carcinoma
Source: BMC Genomics. 2023 Jun 7;24:307. doi: 10.1186/s12864-023-09416-z (PMC10249168; doi:10.1186/s12864-023-09416-z)
Supplement: Supplementary file 1 — Additional file 1: Supplementary Figure 1. Flow diagram of the analysis procedure, including the discovery, training, and validation stages. Supplementary Figure 2. DMG function. A, GO and C, KEGG functional enrichment analyses of DMGs in the promoter regions of genes. B, GO and D KEGG functional enrichment analyses of DMGs in the gene body regions. Supplementary Figure 3. The expression profile of 18-CpG corresponding genes. Supplementary Figure 4. The DNA methylation profile of promoters of 18-CpG corresponding genes. Supplementary Figure 5. Risk scores in the whole TCGA cohort. A, KM survival curve of patients in the high-risk and low-risk groups. The data are shown as median with the interquartile range. Statistical significance was assessed using Log-rank test. The dotted line shows the statistical significance at 50% survival probability. B, Rank of calculated risk score and survival status of high-risk and low-risk patients. The dotted line shows the cutoff value to distinguish ccRCC high-risk and low-risk patients. C, Heat map of methylation levels at 18 CpG sites. D, The 1-, 3-, 5-, and 10-year ROC curves of risk scores. The sensitivity and specificity of this model were determined by the cutoff value. Supplementary Figure 6. Decision curve analyses for overall survival predictions.The colored lines indicate the net benefit of using the model with the combined clinicopathological characters (red), methylation RiskScore (green) and the NomoScore (black). The assumptions that all patients will be alive and that no patients will be dead are represented by grey and black lines, respectively. [file 12864_2023_9416_MOESM1_ESM.zip › Supplementary Figure4.pdf]

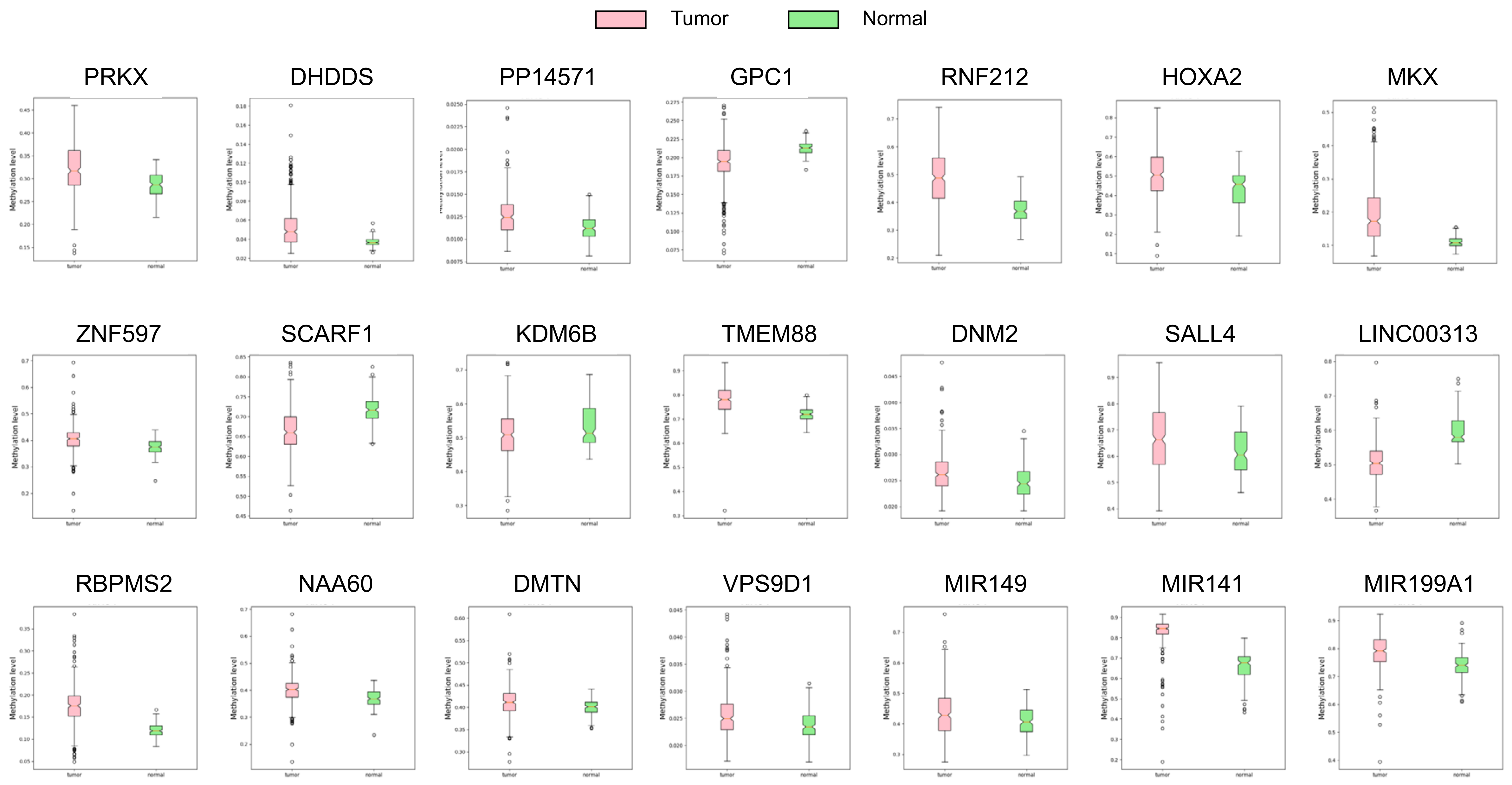

Tumor
Normal
PRKX
DHDDS
PP14571
GPC1
RNF212
HOXA2
MKX
ZNF597
SCARF1
KDM6B
TMEM88
DNM2
SALL4
LINC00313
RBPMS2
NAA60
DMTN
VPS9D1
MIR149
MIR141
MIR199A1
