# Supplementary material for: Identification and validation of a DNA methylation-driven gene-based prognostic model for clear cell renal cell carcinoma
Source: BMC Genomics. 2023 Jun 7;24:307. doi: 10.1186/s12864-023-09416-z (PMC10249168; doi:10.1186/s12864-023-09416-z)
Supplement: Supplementary file 2 — Additional file 2: Supplementary Table 1. Detailed information of the recruited ccRCCpatients. Supplementary Table 2. The univariate Cox regression analysis. Supplementary Table 3. The coefficient of the 18 CpG sites. [file 12864_2023_9416_MOESM2_ESM.docx]

**Supplementary Table 1. Detailed information of the recruited ccRCC patients.**

| **Patients** | **Clinic-pathological characteristics** |
| --- | --- |
| S1  **Gender:** Male  **Age:** 39  **Location:**Left  **Size**: 2.0*2.0 cm | ccRCC, Grade Ⅰ. The nodules (maximum diameter 2.0 cm) are composed of transparent cells. The tumor cells are uniform in size with pyknotic nuclei. The tumor cells are arranged in a solid - glandular tubular shape. There is a fine chicken cage network of blood vessels between the tumor cells. Focal hemorrhage and necrosis are accompanied by cystic changes. |
| S2  **Gender:** Male  **Age:** 45  **Location:** Right  **Size**: 1.5*2.0 cm | ccRCC, Grade Ⅰ. The nodules (maximum diameter 2.0 cm) are composed of transparent cells. the tumor cells are uniform in size, the nuclei are pyknotic, no nucleolus is found, and the heterotypic is not obvious. The cells are arranged into solid - glandular tubular shape. No vascular tumor thrombus and nerve invasion were found. |
| S3  **Gender:** Male  **Age:** 36  **Location:** Left  **Size**: 1.2*1.5 cm | ccRCC, Grade Ⅰ. The nodules are composed of a large number of proliferating transparent cells, the cytoplasm of a small number of cells is eosinophilic, the tumor cells are relatively uniform in size, the nuclei are pyknotic, no nucleolus is found, and the heterotypic is not obvious. The cells are arranged into solid - glandular tubular shape, and there is a fine chicken cage network of blood vessels between tumor cells. Bleeding and cystic changes are seen in local areas. |
| S4  **Gender:** Male  **Age:** 30  **Location:** Left  **Size**: 1.5*2.0 cm | ccRCC, Grade Ⅰ. The nodules are composed of proliferative transparent cells, the tumor cells are uniform in size, the nuclei is pyknotic, and the heterotypic is not obvious. Some of them are solid, and some of the tumor tissue can be seen as hemorrhagic necrosis and cystic transformation. Glomerular hyperemia and interstitial inflammatory cell infiltration were seen in peripheral renal tissue. The perirenal adipose tissue is normal. |
| S5  **Gender:** Male  **Age:** 33  **Location:** Right  **Size**: 5.0*6.0 cm | ccRCC, Grade Ⅰ. The nucleus is ovoid, slightly different in size. some cell nucleoli can be seen, the cytoplasm is transparent or with small red stained particles, arranged in acinar or bubble nest shape, some of them form expanded vesicles with red stained liquid retention, and fungal colonies can be seen. |
| S6  **Gender:** Male  **Age** 57  **Location:** Right  **Size**: 3.0*3.0 cm | ccRCC, Grade Ⅰ. The nodules are composed of proliferative transparent cells, the tumor cells are uniform in size, the nuclei is pyknotic, and the heterotypic is not obvious. Cell are arranged into solid - glandular tubular shape. There is a fine chicken cage network of blood vessels between the tumor cells. Focal hemorrhage and necrosis are accompanied by cystic changes. |
| S7  **Gender:** Male  **Age:** 48  **Location:** Right  **Size**: 2.0*3.0 cm | ccRCC, Grade Ⅰ. The nodules are composed of a large number of transparent cells, the tumor cells are relatively uniform in size, the nuclei is pyknotic, and the heterotypic is not obvious. Cell are arranged into solid - glandular tubular shape. There is a fine chicken cage network of blood vessels between the tumor cells. The perirenal adipose tissue is normal. |
| S8  **Gender:** Male  **Age:** 53  **Location:** Right  **Size**: 2.0*3.0 cm | ccRCC, Grade Ⅰ. The nodules are composed of a large number of proliferating transparent cells, the tumor cells are uniform in size, the nuclei are pyknotic, and the heteromorphism is not obvious. Cell are arranged into a tubular structure of glands, some are solid, some are dilated into cysts, and some of the tumor tissue can be seen with hemorrhage, necrosis and cystic transformation. |
| S9  **Gender:** Male  **Age:** 25  **Location:** Left  **Size**: 2.0*2.0 cm | ccRCC, Grade Ⅰ. The nodules are composed of a large number of transparent cells, showing solid, strip-shaped and glandular tubular structures. The tumor cells are of the same size, and the cell atypia is not obvious. |
| S10  **Gender:** Male  **Age:** 45  **Location:** Left  **Size**: 5.0*6.0 cm | ccRCC, Grade Ⅱ. The renal parenchymal mass is composed of a large number of transparent cell, arranged in a tubular and solid shape of glands. Some of the glandular tubes are dilated in a cystic shape. The tumor nuclei is slightly different in size. Nucleoli can be seen in some cells, and hemorrhage is obvious in some areas. The tumor invades the renal capsule. Some glomerular fibrosis is seen in the renal tissue around the tumor. |

**Supplementary Table 2. The univariate Cox regression analysis**

| **No** | **gene** | **HR** | **95%CI** | **P value** |
| --- | --- | --- | --- | --- |
| **1** | cg15209808 | 3.8 | 1.099-13.138 | 0.035 |
| **2** | cg19871388 | 6.49 | 1.758-23.954 | 0.005 |
| **3** | cg14160518 | 3.683 | 1.296-10.468 | 0.014 |
| **4** | cg01025836 | 0.285 | 0.106-0.768 | 0.013 |
| **5** | cg23885932 | 0.277 | 0.09-0.857 | 0.026 |
| **6** | cg13965612 | 0.072 | 0.007-0.704 | 0.024 |
| **7** | cg09495643 | 0.355 | 0.129-0.975 | 0.044 |
| **8** | cg06747432 | 3.933 | 1.188-13.023 | 0.025 |
| **9** | cg00869668 | 0.037 | 0.002-0.633 | 0.023 |
| **10** | cg02413874 | 0.125 | 0.027-0.582 | 0.008 |
| **11** | cg10725861 | 7.25 | 1.686-31.17 | 0.008 |
| **12** | cg13105599 | 4.805 | 1.274-18.122 | 0.02 |
| **13** | cg03933495 | 0.021 | 0.001-0.538 | 0.02 |
| **14** | cg23067082 | 0.226 | 0.067-0.763 | 0.017 |
| **15** | cg14482313 | 11.041 | 1.568-77.735 | 0.016 |
| **16** | cg05852143 | 4.147 | 1.054-16.323 | 0.042 |
| **17** | cg06303238 | 3.023 | 1.043-8.762 | 0.042 |
| **18** | cg19284039 | 5.45 | 1.948-15.245 | 0.001 |
| **19** | cg25512683 | 0.007 | 0-0.144 | 0.001 |
| **20** | cg19211915 | 7.926 | 1.637-38.379 | 0.01 |
| **21** | cg03306615 | 6.327 | 1.816-22.048 | 0.004 |
| **22** | cg21655830 | 10.755 | 2.166-53.394 | 0.004 |
| **23** | cg09348985 | 0.016 | 0.001-0.403 | 0.012 |
| **24** | cg20348858 | 50.49 | 2.375-1073.45 | 0.012 |
| **25** | cg00499822 | 0.075 | 0.012-0.474 | 0.006 |
| **26** | cg03021802 | 3.651 | 1.032-12.923 | 0.045 |
| **27** | cg06941557 | 1366.947 | 1.156-1616380.542 | 0.045 |
| **28** | cg25598840 | 0.111 | 0.028-0.442 | 0.002 |
| **29** | cg13762320 | 7.75 | 2.079-28.884 | 0.002 |
| **30** | cg14537533 | 12.617 | 2.487-64.02 | 0.002 |
| **31** | cg01286935 | 0.002 | 0-0.106 | 0.002 |
| **32** | cg03411082 | 0.102 | 0.023-0.446 | 0.002 |
| **33** | cg06769202 | 0.22 | 0.057-0.852 | 0.028 |
| **34** | cg26339504 | 7.517 | 1.244-45.436 | 0.028 |
| **35** | cg03744763 | 0.198 | 0.061-0.646 | 0.007 |
| **36** | cg23185774 | 9.402 | 1.441-61.326 | 0.019 |
| **37** | cg05921905 | 0.285 | 0.1-0.812 | 0.019 |
| **38** | cg04499011 | 7.257 | 1.563-33.69 | 0.011 |
| **39** | cg23462514 | 2.387 | 1.039-5.485 | 0.04 |
| **40** | cg18646207 | 5.833 | 1.084-31.381 | 0.04 |
| **41** | cg22190721 | 5.29 | 1.08-25.904 | 0.04 |
| **42** | cg02297063 | 0.376 | 0.148-0.957 | 0.04 |
| **43** | cg02364236 | 5.337 | 1.146-24.843 | 0.033 |
| **44** | cg00508855 | 3.413 | 1.107-10.526 | 0.033 |
| **45** | cg27569446 | 4.629 | 1.048-20.444 | 0.043 |
| **46** | cg16238993 | 0.355 | 0.13-0.966 | 0.043 |
| **47** | cg17367832 | 4.255 | 1.014-17.853 | 0.048 |
| **48** | cg13354988 | 13.816 | 1.308-145.925 | 0.029 |
| **49** | cg03719128 | 23.748 | 1.375-410.184 | 0.029 |
| **50** | cg02164574 | 0.331 | 0.133-0.825 | 0.018 |
| **51** | cg01408486 | 0.205 | 0.055-0.758 | 0.018 |
| **52** | cg22317846 | 0.101 | 0.015-0.676 | 0.018 |
| **53** | cg02311725 | 0.151 | 0.032-0.728 | 0.018 |
| **54** | cg19766489 | 4.192 | 1.439-12.211 | 0.009 |
| **55** | cg03178838 | 0.292 | 0.116-0.737 | 0.009 |
| **56** | cg06577005 | 0.145 | 0.025-0.836 | 0.031 |
| **57** | cg04250451 | 3.221 | 1.021-10.161 | 0.046 |
| **58** | cg02297801 | 0.051 | 0.007-0.37 | 0.003 |
| **59** | cg02979457 | 0.133 | 0.035-0.511 | 0.003 |
| **60** | cg00520933 | 75.746 | 4.294-1336.303 | 0.003 |
| **61** | cg18210365 | 7.027 | 1.946-25.382 | 0.003 |
| **62** | cg04312620 | 6.3 | 1.841-21.567 | 0.003 |
| **63** | cg17868751 | 2.508 | 1.003-6.271 | 0.049 |
| **64** | cg10502957 | 3.503 | 1.005-12.213 | 0.049 |
| **65** | cg11806528 | 14.541 | 3.474-60.86 | 0 |
| **66** | cg16316162 | 42.89 | 6.034-304.85 | 0 |
| **67** | cg14947429 | 39.167 | 8.522-180.018 | 0 |
| **68** | cg16656895 | 65.024 | 7.41-570.555 | 0 |
| **69** | cg21063716 | 14.994 | 3.384-66.448 | 0 |
| **70** | cg26051413 | 6.987 | 2.616-18.66 | 0 |
| **71** | cg18900591 | 9.014 | 2.826-28.745 | 0 |
| **72** | cg24332577 | 12.307 | 3.125-48.468 | 0 |
| **73** | cg25570495 | 12.64 | 3.387-47.172 | 0 |
| **74** | cg26728517 | 21.237 | 5.942-75.896 | 0 |

**Supplementary Table 3. The** **coefficient of the 18 CpG sites.**

| CpG site | coefficient |
| --- | --- |
| cg13965612 | -2.86 |
| cg00869668 | -4.622 |
| cg03933495 | -4.536 |
| cg23067082 | -3.039 |
| cg06303238 | -6.233 |
| cg21655830 | 2.322 |
| cg03021802 | 2.276 |
| cg06941557 | 8.241 |
| cg25598840 | -2.277 |
| cg01286935 | -5.848 |
| cg23462514 | 1.256 |
| cg17367832 | 2.538 |
| cg06577005 | -3.615 |
| cg18210365 | 2.134 |
| cg17868751 | 1.502 |
| cg14947429 | 3.667 |
| cg24332577 | 6.608 |
| cg26728517 | 1.895 |
